# Draft Genome Sequence of *Erwinia mallotivora* BT-MARDI, Causative Agent of Papaya Dieback Disease

**DOI:** 10.1128/genomeA.00375-14

**Published:** 2014-05-08

**Authors:** R. Ahmad Redzuan, N. Abu Bakar, L. Rozano, R. Badrun, N. Mat Amin, M. F. Mohd Raih

**Affiliations:** a Biotechnology Research Centre, Malaysian Agricultural Research and Development Institute (MARDI) Headquarters, Persiaran MARDI-UPM, Serdang, Selangor, Malaysia; b School of Biosciences and Biotechnology, Faculty of Science and Technology, Universiti Kebangsaan Malaysia, Bangi, Selangor, Malaysia

## Abstract

*Erwinia mallotivora* was isolated from papaya trees infected with dieback disease, which were planted at the Malaysian Agricultural Research and Development Institute (MARDI), Malaysia. Here, we report a draft genome sequence of *E. mallotivora* BT-MARDI, which offers an important source of information for understanding pathogen and host interaction during papaya dieback development.

## GENOME ANNOUNCEMENT

*Erwinia mallotivora* is a Gram-negative bacterium classified as a member of the *Enterobactericeae* family (1). *E. mallotivora* was first reported as the causal agent of bacterial leaf spot in *Mallotus japonicas* in Japan (2). In recent studies that included phenotypic observations, biochemical analysis, and genotypic information, *E. mallotivora* was identified as a close relative of *E. papayae* and the causal agent of the papaya dieback disease in Malaysia (3, 4). Thus, it is of great interest to uncover the genome sequence of the isolate obtained from papaya trees infected with dieback. Here, we report the draft genome sequence of *E. mallotivora* BT-MARDI isolated from papaya infected with dieback disease at the Malaysian Agricultural Research and Development Institute (MARDI), Malaysia.

*E. mallotivora* BT-MARDI was identified using Gram stain and biochemical tests. For biochemical tests, an API 20E kit (Biomerieux SA Co., France) was used to confirm the identity of this bacterium. The identity of this particular bacterium was also confirmed using 16S rRNA gene cloning. The genome was sequenced using Illumina and Solexa HiSeq 2000 technology (Illumina, San Diego, CA). The quality of the sequencing output was determined using FastQC. The sequencing data were generated from a paired-end, 101-bp run. The sequencing produced an output of 53,801,258 reads with 550× coverage. FastQC revealed the average G+C content of this bacterium to be about 52.4%. The G+C contents of other reference genomes are 53.6%, 53.4%, 55%, and 53.4% for *E. amylovora*, *E. pyrofiliae*, *E. bilingiae*, and *E. tasmaniensis*, respectively. After trimming, 28,912,327 reads were produced, with 54% trimming efficiency. The reads were assembled (*de novo* assembly) and scaffolded using MSR-CA assembler (5). A total of 80 contigs were produced, which covered a total genome size of 4,824,879 bp.

Fifty scaffolds were generated and remapped against the raw sequence (101 bp) to validate the assembly results. The total numbers of gaps predicted was 50. The annotation was performed using Glimmer3 and aligned to the UNIPROT database using Blastx. The annotation results were validated using Rapid Annotations using Subsystems Technology (RAST) (6). The scaffolds were annotated to have 498 subsystems, 4,855 coding sequences, 4,136 protein-encoding genes, and 81 RNAs. The draft genome sequence of *E. mallotivora* BT-MARDI showed differences from the reference sequences of other *Erwinia* spp. Based on these annotated results, several putative virulence genes have been identified in the genome of *E. mallotivora* BT-MARDI, which include genes involved in secretion systems, sucrose metabolism, capsular polysaccharides, clustered regularly interspaced short palindromic repeats (CRISPRs), iron acquisition and metabolism, motility and chemotaxis, and cell regulation and signaling. These in turn will offer important information to increase our understanding of the host-pathogen interaction during dieback infection in papaya plants.

### Nucleotide sequence accession numbers.

The genome sequence of *E. mallotivora* BT-MARDI has been deposited in DDBJ/EMBL/GenBank under accession number JFHN00000000. The version described in this paper is version JFHN0100000000.
